# Correlation of Positive Blood Cultures with Peripherally Inserted Central Catheter Line Infection in Oncology Patients

**DOI:** 10.7759/cureus.12858

**Published:** 2021-01-22

**Authors:** Christina Platanaki, Nicholas Zareifopoulos, Maria Lagadinou, Konstantinos Tsiotsios, Dimitrios Velissaris

**Affiliations:** 1 Department of Internal Medicine, General University Hospital of Patras, Patras, GRC

**Keywords:** picc, central venous catheter, sepsis, infection, critical care, central line-associated infections (clabsi), coagulase-negative staphylococci, febrile neutropenia

## Abstract

Introduction: The use of peripherally inserted central catheter (PICC) lines offers several advantages compared to traditional central venous catheters (CVCs) as the insertion procedure is minimally invasive, they may be retained safely for longer periods of time, and their use is associated with fewer catheter-related infections. Their use in patients suffering from a malignant disease is common but may pose a greater risk of complications due to the severe immunosuppression associated with treatment. This study was conducted to evaluate the safety of PICC lines in this group.

Methods: This was a retrospective study of oncology patients being treated in a Mediterranean tertiary center. Patients with PICC lines were enrolled in the study if a positive blood culture necessitated the removal of the PICC and subsequent culture of the PICC tip. A comparison was conducted between patients with positive and negative PICC cultures.

Results: Thirty patients were included, four of whom had a positive PICC culture. The most commonly isolated pathogens were coagulase-negative Staphylococci and Corynebacteria. No statistically significant difference was noted in white blood cell (WBC) counts, C-reactive protein (CRP), and Michigan PICC central line associated bloodstream infection (MPC) score between the two groups. Staphylococcus epidermidis was the most commonly isolated pathogen.

Discussion: Though limited by a small sample size and the retrospective design, the findings of this study seem to corroborate existing literature on the subject which suggests that the use of PICC lines in oncology patients is feasible and does not pose unacceptable risk. Further research is indicated to determine subgroups which may be at greater risk of PICC related infections.

## Introduction

Oncology patients may be immunosuppressed due to the underlying illness or as a result of chemotherapy. In addition, they are frequently subjected to invasive procedures and may require prolonged hospitalization during the course of their disease. For these reasons, they are particularly prone to bacterial infection and sepsis, which is implicated in the high mortality rate observed in this population [1].

The presence of central lines is advantageous for the administration of chemotherapy, intravenous antibiotic therapy, and red blood cell or platelet transfusions. In addition, it is necessary for the administration of parenteral nutrition or large volumes of fluid which may be occasionally indicated as standard supportive care.

For this purpose, the use of peripherally inserted central catheter (PICC) lines has been established in recent years as a safe and convenient method of central venous access. PICC lines are special, flexible long tubes that are inserted in a peripheral vein, usually in the upper extremity and are advanced under imaging guidance until the upper tip of the catheter reaches a central vein near the heart, usually the superior vena cava [2]. They offer both the advantage of peripheral intravenous catheters since their insertion requires only the simple puncture of a peripheral vein of the superficial network of the upper extremity, and the advantage of central venal catheters since the tip is inserted in a central vein (superior vena cava).

They can remain inside the human body for up to three months. The mode of their insertion is minimally invasive with very few complications, although rarely it has been associated with bacteremia, as the insertion procedure may introduce pathogens directly into the bloodstream if not performed under sterile conditions [3]. Infection of the catheter is another complication that may arise during the course of treatment, as the catheter may be colonized by biofilm-producing skin flora (especially Staphylococcus epidermidis) which may then be introduced into the bloodstream during use of the PICC. Errors during the handling of the PICC catheter during use may also introduce nosocomial pathogens into the bloodstream [2].

Recently, the use of the Michigan PICC central line associated bloodstream infection (CLABSI) score, the Michigan PICC CLABSI score (MPC) score, has been established as a tool in order to predict the risk of PICC line infection in patients with bacteremia [4].

This study was intended to evaluate the safety of PICC use in oncology patients with solid tumors and/or metastatic disease, who due to the nature of the disease and its treatment may obtain substantial benefit from PICC placement which possibly outweighs the risks despite a high MPC score.

## Materials and methods

This was a retrospective study in which the hospital records were screened for eligible subjects. Patients were enrolled in the study if a) they had a confirmed diagnosis of a malignancy and were admitted to the Department of Internal Medicine of the General University Hospital of Patras, Greece during the study period of nine months (May 2019 - December 2019), b) a PICC line was in place during the course of hospitalization, c) a positive blood culture necessitated the removal and culture of the PICC line and d) not under any medication that affected the number of white blood cells (WBCs). Patients were to be excluded if they had an obvious focus of infection apart from the PICC (a chest X-ray positive for pneumonia, a positive urine culture, a confirmed abdominal infection or findings indicative of a soft tissue infection on physical exam as documented on the chart). The institutional ethical board review requirement was waived due to the retrospective nature of the study. Thirty patients fulfilling the aforementioned criteria were enrolled. In the included patients, one or more positive blood cultures were obtained while they were in the inpatient department, which led to the removal of the PICC and to culture of the tip as per institutional protocol. A complete blood count, basic metabolic panel, C-reactive protein (CRP) concentration, chest X-ray, and urine cultures were also obtained on the day of PICC removal as this is the standard workup procedure in the institution. The MPC score for patients, as well as their basic demographic information (age, sex), were also recorded from the chart. WBC count, CRP, time since the PICC was inserted, and MPC scores were compared by non-parametric Mann-Whitney test between patients with positive PICC cultures and those with negative cultures of the PICC tip. Statistical Package for the Social Sciences, version 25 (SPSS Inc., Chicago, IL) was used for statistical analysis. The pathogens identified in cultures were also recorded.

## Results

Of the 30 patients included in total, 16 (53.3%) were males. The mean age of the patients was 57.73 (±16.19) years.

As far as the health indices are concerned, the mean of the WBC was 2558.57 (±2415.83), the neutrophil count was 1754.73 (±2077.36) and CRP was found equal to 6.80 (±8.52). Finally, the mean time that the PICC had been in place was 75.03 (±46.27) days. The results are presented in Table 1.

**Table 1:** Demographic data and health indices (N=30)

Table 1: Demographic data and health indices (Ν=30).	
Variable	Result [mean (standard deviation)]
Age	57.73 (16.19)
Wight blood cell count	2558.57 (2415.83)
Neutrophil count	1754 (2077.36)
C-reactive protein	6.80 (8.52)
Time after PICC placement (days)	75.03 (46.27)
MPC score	4.20 (1.32)
PICC- Peripherally inserted central catheter, MPC- Michigan PICC central line associated bloodstream infection	

The most frequent pathogen isolated in the blood cultures was Staphylococcus epidermidis (50%), while Staphylococcus hominis (26.7%) and Staphylococcus haemolyticus (16.7%) were also common. These results are presented in Table 2.

**Table 2:** Results of blood cultures

Table 2: Results of blood cultures.		
Type of pathogen	No	%
Staphylococcus epidermidis	15	50.0
Staphylococcus hominis	8	26.7
Corynebacterium species	7	23.3
Staphylococcus haemolyticus	5	16.7
Acinetobacter baumannii	3	10.0
Klebsiella pneumoniae	3	10.0
Pseudomonas aeruginosa	3	10.0
Bacillus megaterium	1	3.3
Candida parapsilosis	1	3.3
Escherichia coli	1	3.3
Micrococcus luteus	1	3.3
Morganella morganii	1	3.3
Staphylococcus aureus	1	3.3
Streptococcus sanguinis	1	3.3
Total of different microbes isolated from the 30 patients	51	

Only four out of the 30 patients with a positive blood culture had also a positive culture of the tip of the PICC (13.3%). The results from the PICC cultures are presented in Table 3.

**Table 3:** Results of peripherally inserted central catheter (PICC) line cultures

Table 3: Results of PICC line cultures		
	Νo	%
Negative culture	26	86.7
Positive culture	4	13.3
Acinetobacter baumannii / Pseudomonas aeruginosa	1	3.3
Staphylococcus aureus	1	3.3
Staphylococcus epidermidis	1	3.3
Staphylococcus coagulase- Negative	1	3.3

As far as the results of the laboratory examinations were concerned, no statistically significant difference was identified between patients with positive and negative culture of the PICC line. The results for the time since the PICC was placed were also similar (Table 4).

**Table 4:** Comparison of continuous variables between patients with positive and negative PICC culture

Table 4: Comparison of continuous variables between patients with positive and negative PICC culture			
Variable	Positive PICC culture (N=4) [Mean (Standard deviation)]	Negative PICC culture (N=26) [Mean (Standard deviation)]	p-value*
Age	60.50 (20.20)	57.31 (15.92)	0.62
Wight blood cell count	4612.50 (5746.33)	2242.58 (1424.35)	0.93
Neutrophil count	3636.00 (5098.57)	1465.31 (1110.43)	0.98
C-reactive protein	14.65 (16.65)	5.60 (6.29)	0.58
Time after PICC placement (days)	75.385 (48.98)	72.75 (26.32)	0.66
MPC score	4.500 (1.73)	4.154 (1.29)	0.79
* Non-parametric Mann-Whitney test			
PICC- Peripherally inserted central catheter, MPC- Michigan PICC central line associated bloodstream infection			

Due to the very low number of positive PICC cultures, the statistical power of the comparison was minimal. From the observation of the variables in Table 4, a weak trend of mildly elevated values is observed in the patients with positive culture in comparison with the patients with negative culture, but this comparison cannot be strongly supported.

## Discussion

The findings of our study indicated that despite the presence of PICC lines in this high-risk group of immunocompromised patients, infection of the PICC tip was uncommon. Furthermore, a positive PICC tip culture does not definitely prove an invasive central line infection especially in the absence of clinical signs of sepsis, as in a much larger cohort of cancer patients both exit site infections and noninvasive catheter colonization were at least eight times as common as invasive central catheter-associated infections [5]. These findings may provide preliminary evidence that the use of PICC lines may be justified in this high-risk patient group. This is supported by a previous study which found a low overall risk of complications in cancer patients who bore a PICC, which was however greater for overweight individuals [6].

There are many studies demonstrating that the occurrence of CLABSI is more frequent in patients with intensive care unit stays and patients with malignant disease with a PICC line. These varying data raise the question of whether PICCs are truly safer than central venous catheters (CVCs) with respect to catheter-associated bloodstream infections [7,8]. A meta-analysis of 23 studies and 57250 patients demonstrated a lower risk of CLABSI with PICCs compared with standard CVCs, though the advantage of PICC lines may be limited to outpatients, as in hospitalized patients when adjusting for the period of time the line has been in place, no difference was found [9]. In 2019, Krein et al. [10] conducted a multicenter prospective cohort study of the 70-day follow-up period of 438 patients who had received a PICC line. Over half (61.4%) of the patients reported signs of at least one potentially serious complication such as bloodstream infection (17.6%) or deep vein thrombosis (30.6%). Though early studies showed that catheter-related bloodstream infection rates in PICC lines were significantly lower than in CVCs, recent evidence suggests little difference between the two [8]. Another study examined the occurrence of CLABSI in patients with hematologic malignancies. The feasibility and safety of PICC for use in acute myeloid leukemia was recorded in 89 patients. The PICC increased the quality of life in these patients during the chemotherapy period. Bacteremia in patients with PICCs was comparable to that of other IV lines, suggesting that PICC use is feasible in this patient population [11].

Patients with malignancies are likely to require central line placement during treatment, and peripherally inserted catheters are increasingly used in contemporary medicine because of their characteristics of feasibility, accessibility, safety, versatility, and cost-effectiveness [2]. CLABSIs are one of the most serious complications of PICC use and are associated with increased morbidity and mortality rates. The reported incidence of PICC CLABSI has ranged from 1.0-7.71 per catheter days, with an estimated mortality risk οf 12%-36% [12]. In a cohort of 746 hospitalized patients, factors associated with increased risk for PICC-related infection were the use of the PICC for chemotherapy administration, prolonged hospitalization, and use of a double-lumen device [7]. In a cohort of cancer patients, the most common complications resulting in PICC removal was upper extremity thrombosis, followed by exit site infection and catheter occlusion, with a relatively lower rate of CLABSI [13]. In a study comparing PICC lines with implantable ports (another form of central venous access commonly used in this patient population), PICC lines were associated with a higher risk of complications which was attributed mostly to a significantly higher risk of thromboembolic events [14].

In this study, PBSI occurred in 30 of 480 oncology patients treated in our tertiary center for whom a PICC line was inserted, resulting in an infection rate of 6.25% for that short period of nine months. This PBSI rate is very close to those previously reported [4]. The reason for such a proportion may be the study setting, as all participants were hospitalized patients who are more susceptible to any form of hospital-acquired infection. Chopra et al.’s meta-analysis found a tenfold greater risk of CRBSI among hospitalized patients (5.2%) than among outpatients who received PICCs [9].

In our study, the median duration of PICC was approximately 75 days. Patients with positive PICC cultures have had the catheter for a longer time than those without any isolation from PICC cultures. Al Raiy et al. reported a 2.3 per 1000 catheter-days incidence of CRBSI with the median time to development of infection 23 days in their prospective study [3]. Studies of PICC infections in cancer patients are few. Chopra et al.’s meta-analysis found rates of PICC-associated CRBSI similar for patients with hematologic malignancies and other types of cancer, those who were critically ill, and those requiring total parenteral nutrition [9].

Another large cohort study was conducted using data from the Michigan Hospital Medicine Safety consortium [15]. Of the 23,088 patients who bore PICCs during the study period, 1.1% developed CLABSI. Among the significant risk factors associated with PICC-CLABSI were hematological malignancies. Moreover, the MPC was significantly associated with risk of CLABSI (p<0.0001). Our study which included the MPC score did not show a statistically significant correlation between the incidence of bacteremia and the MPC score.

The present study has several limitations, as the study period was short and few patients were ultimately enrolled, leading to diminished statistical power. For this reason, it was not possible to establish statistically significant correlations between the measured data and infection of the PICC tips. Furthermore, the retrospective nature of the study may be a potential source of bias. We did not require positive follow up blood cultures in the inclusion criteria as the prompt initiation of antibiotic treatment would be expected to result in all subsequent cultures being negative, but given that normal skin flora (Staphylococcus epidermidis) was among the most commonly isolated pathogens, the possibility of initial false-positive culture results cannot be excluded.

## Conclusions

Our study, although underpowered, provides some preliminary evidence that in patients with malignant disease who bear a PICC line, a positive blood culture does not directly implicate the PICC as the cause. Especially in patients where multiple peripheral venipunctures are required, or who apart from the PICC also bear additional peripheral venous catheters, additional research is required to determine which of these poses a greater risk for bloodstream infections, specifically adequately powered prospective cohort studies of the same population. The limitations mentioned previously may limit the generalizability of this study to clinical practice, although the literature is in favor of the use of PICC lines in oncology patients. Our study sheds some light on the pathogens that may be implicated in bloodstream infections in this population which may influence empirical antibiotic regimens at least in the area of Western Greece where the cohort was based.
